# Evolution of the Canadian regulatory system for plant biotechnology

**DOI:** 10.3389/fbioe.2026.1912331

**Published:** 2026-09-18

**Authors:** Luis Luque, Jennifer Hubert

**Affiliations:** CropLife Canada, Ottawa, ON, Canada

**Keywords:** agriculture, biotechnology, genome editing, new genomic techniques (NGTs), plant breeding innovations (PBI), policy, regulatory framework, sustainable agricultural applications

## Abstract

Canada was one of the first countries to adopt agricultural biotechnology products. This has served Canada well as it is constantly ranked among the world’s top agriculture commodities exporters with its products reaching markets in all continents. However, the speed at which regulations enabling the commercialization and adoption of the latest agricultural biotechnologies did not match the pace with advancements in the field. As the years went by, a lack of clarity about regulatory interpretation created an unlevel playing field for Canadian plant breeders and companies. This started to change in 2022 when new policy changes were announced by the Canadian government, paving the way for the adoption of the latest technologies. The aim of this document is to bring together the key pieces of legislation and policy elements governing plant breeding innovations in Canada into a single consolidated reference. The paper reflects perspectives and interpretations by Canadian industry regulatory experts on the 38-year history and evolution of the regulatory landscape in Canada to support clarity and consistent application for future developers and innovators in this sector.

## 1 Introduction

Canada’s agriculture and agri-food industry is an integral part of the Canadian economy and global food security. In Canada, the sector contributes $149.2 billion (7% of GDP) and employed 2.3 million (1 in 9 jobs) in 2024 (AAFC, 2025a) Sustaining this contribution in the face of climate pressures, evolving consumer demands, and global competition depends in part on the timely development and deployment of innovative crop varieties. However, as evidenced in a recent report from the Canadian Agri-food Policy Institute (CAPI, 2025), regulatory barriers and delays are hindering the sector from reaching its maximum potential (CAPI, 2025).

Regulatory frameworks play a central role in shaping the pace, direction, and feasibility of crop innovation. The scope, predictability, and proportionality of pre-market requirements influence not only development timelines and costs, but also which traits and technologies are prioritized by developers (Smyth et al., 2014; Whelan et al., 2020). As plant breeding technologies, including gene editing and other precision methods, enable increasingly targeted and predictable modifications (Chen et al., 2019), the need for clarity on how these and other products will fit within existing regulatory frameworks is critical.

Humans have been improving crops for thousands of years to address their unique demands. However, the techniques used to achieve these objectives have evolved dramatically over the past century with breeders gaining access to increasingly sophisticated tools for generating, identifying, and selecting genetic variation (Enfissi et al., 2021; Singer et al., 2021). Plant biotechnology is an umbrella term that can encompass a wide range of methods used by plant breeders to introduce, select, and combine genetic variation to develop improved crop varieties. These can include conventional breeding methods, such as crossing, selection, and mutagenesis; modern biotechnology-based methods, including recombinant DNA (rDNA) techniques used to introduce specific genetic material; and gene editing and other precision breeding techniques, which can make targeted changes to a plant’s existing genetic material (Lusser et al., 2011; Health Canada, 2022d; AAFC, 2025b).

While biotechnology is often defined broadly, it is commonly associated with modern biotechnology techniques like genetic engineering and rDNA methods. Whereas the industry has more recently begun to use the term plant breeding innovations (PBI) to describe all the evolving ideas and practices to improve plant breeding both old and new (CropLife Canada, 2026; ISF, 2026; Seeds Canada, 2026). New breeding techniques (NBTs), New genomic techniques (NGTs) and Precision Breeding Techniques (PBTs) are a subset of tools and methods enabling more precise, efficient, and targeted genetic changes in plants (EU Commission, 2026). Consequently, when plant breeders utilize these new techniques, they are able to develop plant varieties more precisely and rapidly (ISF, 2026). Although these approaches differ in how genetic variation is generated or introduced, they share the same fundamental objective: developing plants with desirable characteristics such as improved yield, disease or pest resistance, stress tolerance, nutritional quality, or other agronomic traits. Importantly, the method used to develop a plant does not, in itself, determine whether the resulting product presents a novel safety characteristic (Health Canada, 2022d; CFIA, 2023c).

Canada’s regulatory system for plant biotechnology stands out globally because it focuses on the characteristics of the final product. Regardless of the methods used in plant breeding (whether conventional techniques, biotechnology or gene editing) products may require pre-market assessment if they are considered “novel” (see novelty definitions provided in Table 1). This product-based approach is unique within the global regulatory landscape as many jurisdictions have adopted more “process-based” approaches where products trigger pre-market oversight based on the breeding method used to develop the product, particularly where the product meets the definition of a Living Modified Organism (LMO) under the Cartagena Protocol on Biosafety. Under Article 3 of the Protocol, an LMO is defined as any living organism that has a new combination of genetic material where genetic material has been introduced using *in vitro* nucleic acid techniques that overcome natural physiological reproductive or recombination barriers and are not techniques used in traditional breeding and selection (modern biotechnology) (UN CBD, 2000). This includes organisms containing recombinant DNA (rDNA) or those that are transgenic. Consequently, many jurisdictions broadly capture products developed using these technologies within their regulatory frameworks, regardless of the characteristics of the final product (Lubieniechi et al., 2025). In general usage, LMOs are often referred to as Genetically Modified Organisms (GMOs), although the term GMO is not consistently defined and can have different meanings across jurisdictions and contexts. Canada ‘s product-based regulatory approach offers some advantages, particularly in its potential adaptability as new plant breeding technologies emerge and are adopted by the sector.

**TABLE 1:** Relevant agencies, legislation, guidance and novelty definitions for the authorization of unconfined release of novel plant breeding innovations and corresponding documents.

Department	CFIA – PBO	CFIA – AFD	Health Canada
Governing Act and Regulation	*Seeds Act* (Government of Canada, 1985c) *Seeds Regulation*, Part V (Government of Canada, 1996)	*Feeds Act* (Government of Canada, 1985a) *Feeds Regulation* (Government of Canada, 2024)	*Food and Drugs Act* (Government of Canada, 1985b) *Food and Drugs Regulation*, Division 28 Part B (Government of Canada, 1999)
Product regulated	Plants with novel traits	Novel Feed	Novel Food
Regulatory policy directives	Directive 2009-09: Plants with Novel Traits Regulated under Part V of the *Seeds Regulations*: Guidelines for Determining when to Notify the CFIA (CFIA, 2026c) Directive 94-08: Assessment criteria for determining environmental safety of plants with novel traits (CFIA, 2017)	RG-1 Regulatory guidance: Chapter 2 - Data requirements for single ingredient approval and feed registration 2.6 Guidelines for the assessment of novel feeds: Plant sources (CFIA, 2024b)	Guidelines for the safety assessment of novel foods (2006 updated in 2022′) (Health Canada, 2022d)
Novel trait Definition	(a) has been intentionally selected, created or introduced into a distinct, stable population of cultivated seed of the same species through a specific genetic change, and (b) based on valid scientific rationale, is not substantially equivalent, in terms of its specific use and safety both for the environment and for human health, to any characteristic of a distinct, stable population of cultivated seed of the same species in Canada, having regard to weediness potential, gene flow, plant pest potential, impact on non-target organisms and impact on biodiversity; (*caractère nouveau*)	(a) has been intentionally selected, created or introduced into the feed through a specific genetic change; and (b) based on valid scientific rationale, is not substantially equivalent, in terms of its specific use and safety for human or animal health or the environment, to any characteristic of a similar feed that is listed in the *Canadian Feed Ingredients Table*. (*caractère nouveau*)	a food that is derived from a plant, animal or microorganism that has been genetically modified such that: (i) the plant, animal or microorganism exhibits characteristics that were not previously observed in that plant, animal or microorganism, (ii) the plant, animal or microorganism no longer exhibits characteristics that were previously observed in that plant, animal or microorganism, or (iii) one or more characteristics of the plant, animal or microorganism no longer fall within the anticipated range for that plant, animal or microorganism. (*aliment nouveau*)

In Canada, there are three separate regulatory units within two federal government departments that oversee the pre-market assessment and approval of these products – The Canadian Food Inspection Agency (CFIA) oversees environmental release (i.e., plants with novel traits) and animal feed (novel feed) use, and Health Canada (HC) oversees the food (novel food) use. While Canada’s regulatory framework has remained consistent since its inception in 1993, over time there have been several policy updates that have changed specific submission requirements and clarified the pre-market regulatory requirements for different products.

Due to the complex nature of Canada’s pre-market oversight system–spanning multiple *Acts*, Regulations and policies administered across three regulatory units^1^ – and the changes that have occurred over the years, a comprehensive and consolidated description of the framework is not readily available. This fragmentation, combined with evolving guidance and interpretations, can create challenges for a consistent understanding and application by product developers and other stakeholders.

This paper aims to provide a clear and integrated overview of Canada’s recently updated regulatory framework by bringing together the key legislative authorities, regulatory requirements, policy guidance and supporting transparency mechanisms in one place. By situating current requirements within their historical and policy context and providing an additional interpretation and perspective from experienced Canadian industry regulatory experts, it aims to support a consistent understanding of both the structure and intent of the system, thereby facilitating more informed use and application over time. The updated regulatory framework has only recently been implemented, and practical experience with its application remains limited. As a result, some observations regarding the interpretation, implementation, and potential implications of the updated framework are based on the information and experience available to date, and further experience with implementation may provide additional insight. While not the primary focus, the paper also provides industry reception of updated guidance and identifies select opportunities for further refinement of the framework to enhance its effectiveness and make Canada the most competitive regulatory environment for supporting PBI.

## 2 The Canadian regulatory framework for biotechnology products

### 2.1 Historical background: governing laws and regulations

In 1988 the Canadian Agri-Food Research Council held a workshop with leading scientists to discuss the regulation of agricultural biotechnology. A key recommendation from the workshop was that the trigger for government pre-market safety assessment for products of plant breeding should be based on the intended trait rather than the specific technology used to introduce the novel trait to the plant (CFIA, 2007). This ‘product-based approach’ is founded on the premise that any potential risk lies with the final product and its use, rather than the technology used to develop the product. That use includes cultivation, animal feed use, or human food consumption or any combination of the three (Schnell et al., 2015).

In 1993 the Government of Canada refocused its 1983 National Biotechnology Strategy with the introduction of a revised Federal Regulatory Framework for Biotechnology. The national biotechnology strategy was later renewed in 1998 (Industries Canada, 1998; Health Canada, 2017). In the 1993 strategy, Canada adopted a product-based regulatory framework by amending the existing *Seeds Act 1985, Foods and Drug Act 1985 and the Feeds Act 1985* and adding new parts dealing with biotechnology products through respective regulations, Table 1. This approach provided the flexibility needed to leverage existing regulation and expertise while adapting the regulatory system to evolving scientific developments in plant breeding (CFIA, 2016). The *Feed* and *Seed Acts* were later listed under Schedule 4 of the *Canadian Environmental Protection Act* (CEPA) which came into force in 1999. CEPA became the central legislative authority for the government to ensure that new substances (e.g., chemicals, polymers) and some animate products of biotechnology, not already covered by existing *Acts* (CEPA Schedule 4), were assessed for their potential to harm human health or the environment prior to their introduction into the Canadian environment. CEPA is effectively seen as a safety net for products not governed by other acts. *Acts* listed in Schedule 4 are deemed as CEPA equivalent, and products governed under those acts are exempt from notification for approval under CEPA (ECCC, 1999).

Rather than creating new legislation or regulatory bodies, the government built upon its existing regulatory framework to oversee environmental release of plants with novel traits (PNTs), novel animal feed, and novel food. Specific sections were added to existing regulations for seed, feed and food, each with its own unique definitions for novelty. While these definitions are not identical, they are closely aligned, particularly between PNTs and Novel Feeds (see Table 1). To help interpret the regulations, in 1994 HC and the CFIA issued directives or guidance documents which have been updated on multiple occasions over time (Guidelines for the safety assessment of novel foods and Directive 94-08 respectively). Followed shortly in 1995 by the publication of Directive 95-03 for novel feeds. Despite their separate legislative authorities and definitions of novelty, a more consistent interpretative definition was incorporated into the three sets of guiding policies, whereby novelty was most aligned with the regulatory definition in the *Food and Drugs Regulations* 1999, Division 28 Part B. The three policies consistently described a novel product as a plant that has:

- a new characteristic,
- a characteristic that is significantly “outside the anticipated range” for the crop species, or
- a characteristic no longer present that was previously observed.

Under the Canadian regulatory framework, the responsibility to determine whether a product meets the definition of novelty — therefore requiring a pre-market assessment — rests with the product developer (Health Canada, 2022d; CFIA, 2026c). Where there is uncertainty, developers may seek confirmation through a novelty determination process. Once the product is determined to be novel, the regulators also offer a voluntary pre-submission consultation process to provide clarity on submission requirements. Guidance exists to clarify this process (CFIA, 2020). Section 2.4 Historic vs*.* new processes contains further information.

While the three government bodies have unique definitions and governing legislation, in practice, almost all products approved to date have met the novelty criteria and required authorization by all three groups. When a plant is considered a PNT as well as a novel food and a novel feed, regulatory decisions are harmonized, and the CFIA and HC send separate letters to the applicant and publish a decision on their respective websites. This coordinated approach, known informally as the “no split approval” (CFIA, 2019) is intended to minimize the risk of unauthorized products entering the environment, feed chain, or food supply prematurely. This is not meant to imply that all agencies need to complete a review if triggered by one. Albeit rare, there are cases where a conventional product was only deemed novel by one regulatory unit and only needed one approval. Furthermore, in certain cases where a plant known not to grow in Canada, such as rice and cotton, proponents can receive a partial authorization to ensure that in the rare case of an incidental release (i.e., transportation spill) the proponent is in compliance as these risks have been assessed by the governing authority.

While the general framework and regulatory triggers remained consistent over time, the government agencies regularly reviewed processes and data requirements aiming to provide clarity and align requirements with the latest scientific understanding or the history of safe use through changes to policy and guidelines. Where regulations are the official legislation which must follow an extensive rigid process to create or change, policy and guidance documents are resources used to provide interpretations on complex legal language on how to meet regulatory requirements and can be updated more readily (Government of Canada, 2018).

The CFIA Plant Biosafety Office (CFIA-PBO) made the most attempts to modernize their requirements. These efforts can be attributed to the fact that this regulatory office is the only unit with a section dedicated to policy development that is separate from the risk assessment group. Key policy improvements implemented by CFIA include a simplified risk assessment process for re-transformations and re-mutations introduced in 2012 and a streamlined process for ‘similar PNTs’ introduced in 2017, as described in the CFIA directives 94-08 and RG-1 chapter 2 (CFIA, 2017; 2024b).

### 2.2 Industry interpretation, confusion and need for clarification

Canada’s regulatory framework was designed to be product-based, but in practice, implementation has not always fully reflected this original intent. Initially, it was anticipated that not all products developed using rDNA technologies would require pre-market oversight. Rather, it was expected that the focus would be on the specific, intended (novel) change in the plant and its relative safety compared to its conventional (not novel) counterpart. However, this period in the late 20th century coincided with significant global political pressure to impose strict oversight on all plants developed with rDNA and the food/feed derived therefrom, as all products developed with rDNA effectively met the definition of an LMO (Burke, 2004; European Union, 2010; Kuntz, 2014).

International guidelines and reports developed by multilateral bodies like the United Nations Food and Agriculture Organization (FAO) Codex Alimentarius Commission (CODEX) (CODEX, 2003) and the Organization for Economic Co-operation and Development (OECD) (OECD, 1986) aimed at harmonizing safety/risk assessment of rDNA-derived plants and their food products, further reinforced regulatory approaches centered on the potential for unintended effects associated with rDNA breeding methods. In addition, developers would voluntarily submit applications for government approvals–even if not required like it is the case in the USA and Canada–to demonstrate that their products are safe and compliant with the objective to provide added comfort to stakeholders.

As a result, like other jurisdictions implementing more process-based approaches, in practice Canada effectively treated all rDNA-derived plants as novel by default – even though this was not explicitly required by regulations or guidance. Consequently, with one limited exception involving a non-commercial petunia product not intended for environmental release, food, or feed use, all products of rDNA (see LMO definition) to date have been subject to pre-market assessment and approval as PNTs, novel food and novel feeds, totaling 126 LMOs (i.e., transgenics) approved in Canada as of May 2026 (CFIA, 2026d).

At the same time, Canada’s product-based philosophy–the risk lies in the end product rather than the breeding process–also meant that conventionally bred plants could require pre-market authorization if they expressed a novel trait. Consequently, the CODEX safety/risk assessment approach primarily designed for products of rDNA, was extended to conventionally bred varieties that contained novel traits. This set Canada apart from the rest of the world and resulted in more products undergoing assessment under the Canadian regulatory framework than in any other country. To date, 39 non-LMOs (i.e., non-transgenic, induced mutation) have required review and approval, this represents 24% of plants with novel traits (and food/feed derived therefrom) approved to date (CFIA, 2026d). Most of these 39 non-LMOs contained new herbicide tolerant traits (CFIA, 2026d). Even though this is a relatively small number considering the thousands of new conventionally bred plant varieties released globally each year (FAO, 2025) this regulatory scope remains unique internationally.

The challenge, however, has been determining when a product of conventional plant breeding should be considered ‘novel’ for regulatory purposes. While the Canadian government has maintained it will be rare for conventional plant breeding to trigger novelty, the objective of plant breeding is inherently to introduce novelty; therefore, developers often faced significant uncertainty when attempting to determine whether a particular characteristic could trigger regulatory oversight essentially creating a regulatory “black box” of uncertainty. This ambiguity discouraged innovation, with some public breeding programs being scaled back or cancelled altogether due to fears of triggering costly regulatory oversight (Smyth et al., 2020). These lost opportunities affected the diversity of plant varieties available to Canadian farmers.

The case-by-case approach pursued by Canadian regulators did not provide the predictability needed by product developers, and it proved challenging to create explicit rules for quantitative traits that would apply consistently across crop types and geographic contexts. Developers struggled to demonstrate that a trait was not “significantly outside the anticipated range” when such ranges were not defined, were evolving, or represented merely an average of measured values rather than the full spectrum of trait expression.

The emergence of new breeding technologies in the early 21st century, particularly genome editing, further underscored the need for clearer guidance on regulatory scope for products of plant breeding as these technologies enable increasingly precise modifications, often producing outcomes indistinguishable from those achievable through conventional breeding (Martínez-Fortún et al., 2022). Despite, the “product-based” regulatory framework, which in principle is adaptable to such developments, uncertainty regarding the interpretation of novelty criteria increasingly complicated the ability of developers to self-assess their products for novelty.

Recognizing the growing need for regulatory clarity – both to eliminate longstanding uncertainty and respond to emerging biotechnologies – the Government of Canada announced in 2018 that it would take steps to help the agricultural sector innovate, grow, and remain competitive by addressing ambiguity in plant breeding regulations. Such improvements were deemed necessary to enable the sector to maintain a steady supply of nutritious crops and improve agricultural sustainability. Beginning in 2020, federal departments engaged with a broad range of stakeholders to develop rigorous, science-based policy proposals, and public consultations were launched (Health Canada, 2022c; CFIA, 2023e; 2024c) to guide the development of updated guidance and implementation practices.

### 2.3 Updated guidance for plant breeding innovations

Beginning with Argentina in 2015, a number of jurisdictions began clarifying how their existing GMO/LMO regulatory frameworks would apply to products developed using genome editing and other new breeding techniques (Whelan and Lema, 2015; Ricroch et al., 2026). In many cases, these policies established that products without a “novel combination of genetic material”—particularly those in which no foreign DNA remains in the final product—would not fall within the jurisdiction’s GMO/LMO framework, while introducing streamlined or case-by-case procedures or consultative processes to confirm their regulatory status. Other jurisdictions adopted more restrictive approaches, with regulatory status varying according to the type or extent of genetic modification, including distinctions among Site-Directed Nuclease (SDN) 1, SDN-2, and SDN-3 applications (Zannoni, 2019; Government of Australia, 2026; Ricroch et al., 2026). The specific details and differences among various approaches around the world are beyond the scope of this paper and have been discussed extensively elsewhere (Menz et al., 2020; Sprink et al., 2022; Ricroch et al., 2026). This evolving international regulatory landscape, together with advances in genetic tools applied to plant breeding and Canadian regulatory experience, provided important context as Canada considered how its existing product-based framework should apply to new breeding technologies.

To develop the updated guidance the CFIA and HC embarked on a thorough process that included an extensive literature review, reports from international regulatory bodies, webinars with leading experts and public consultation and consideration of regulatory experience gained over the last 30 years (Health Canada, 2022f; CFIA, 2023c). In 2022, after several years of analysis followed by public consultation with agriculture stakeholders, the Government of Canada started to publish updated and clarified guidelines for plants with novel traits, novel foods and novel feeds from plant sources. Updates were limited to adding or replacing existing guidance documents, and no updates to the governing *Acts* or *Regulations* were deemed necessary. A summary of the updates is shown in Table 2.

**TABLE 2:** Summary of updated guidance documents and transparency initiatives.

Year published	Agency	Updated/New guidance documents and initiatives
2022	Health Canada	Scientific opinion on the regulation of gene-edited plant products within the context of Division 28 of the Food and Drug Regulations (Novel Foods) (Health Canada, 2022f) The following appendices were added to Health Canada’s Guidelines for the Safety Assessment of Novel Foods • Appendix 1: Health Canada Guidance on the Novelty Interpretation of Products of Plant Breeding (Health Canada, 2022a) • Appendix 2: Health Canada Guidance on the Pre-Market Assessment of Foods Derived from Re-transformants (Health Canada, 2022b) Launch of transparency initiative for gene-edited plants developed for food use that are not novel foods (Health Canada, 2022e) Launch of list of non-novel products of plant breeding for food use (Health Canada, 2026b)
2023	CFIA (Seed for environmental release)	Rationale for updated guidelines for determining whether a plant is regulated under Part V of the Seeds Regulations (Directive 2009-09)(CFIA, 2023c) Updated/replaced Directive 2009-09: Plants with novel traits regulated under Part V of the Seeds Regulations: Guidelines for determining when to notify the CFIA (CFIA, 2026c)
2024	CFIA (Livestock Feed)	Added appendix III to RG-1 Regulatory guidance (CFIA, 2024a): Chapter 2 - Data requirements for single ingredient approval and feed registration • Appendix III: Guidance on how to determine when a plant-derived ingredient requires a pre-market evaluation
2025	CFIA (Seed)	Launched List of non-novel products of plant breeding for environmental release (CFIA, 2025b)
2026	CFIA (seed)	Revised policy governing herbicide tolerant crops and stewardship plans (CFIA, 2026c)

While each of the guidelines is specific to the relative product type – novel foods, novel feeds, or plants with novel traits – they share several key themes:

- Recognition of an evolving technology landscape.
- Recognition of the need to clarify how products of new breeding techniques such as genome editing fit within the Canadian regulatory framework, in the context of a product-based system.
- A scientific rationale reaffirming the inherent safety of plant breeding.
- Leveraging the accumulated regulatory experience of nearly 30 years of approving biotechnology products.
- Clarification that the presence of foreign DNA in the final plant product results in a novel product that requires pre-market review and approval. This provides a product-based justification for capturing all rDNA products.

#### 2.3.1 Scientific rationale

Along with the updated guidelines, HC and CFIA both published scientific rationales supporting the need and foundation for the updates (Health Canada, 2022f; CFIA, 2023c). Both documents recognize the safe history and evolution of plant breeding tools; how genome edited plant products fit within the product based regulatory approach; and the familiarity and regulatory experience gained after 30 years of evaluating the safety of novel feeds, foods and plants with novel traits with no adverse effects resulting from their commercialization.

HC and the CFIA conducted extensive and separate literature reviews and key scientific findings included:

- Genome editing can result in genetic changes that are similar or identical to conventional breeding, with higher precision and fewer unintended effects.
- The risk profile of rDNA technologies, after almost 50 years of study and application, is now well understood. Unintended effects are no different than those that occur through conventional breeding techniques (Schnell et al., 2015).
- Recognition of universal plant breeding practices that support food, feed and environmental safety regardless of the breeding method used.
- There is no inherent risk associated with any particular plant breeding technology (including genome editing), and regulatory oversight should remain focused on the novelty of the trait and how it could impact safety end points.

Importantly, the updated materials also acknowledge that plant breeding is a rigorous, science-driven process involving selection, in-depth product characterization, and continuous oversight throughout a product’s lifecycle, all of which inherently support food, feed, and environmental safety. Regulators recognize that these practices, applied regardless of the breeding method used, ensure developers understand, manage, and mitigate potential safety risks from development through to commercialization.

While the underlying frameworks and safety principles are consistent across all three governing bodies, each developed unique criteria to determine novelty based on the end point to be protected – food, feed, or environment. These unique criteria are described below.

#### 2.3.2 Health Canada update

In 2022 HC published updated guidelines for novel food products derived from plant breeding, added as Appendix I to the Guidelines for the Safety of Assessment of Novel Foods (Health Canada, 2022d). The new guidelines add to the long-standing health and safety objectives of the existing guidelines, while providing greater clarity and predictability for developers to self-determine if their plant breeding products meet the definition of “novel food” and require pre-market review and approval.

The new Appendix I outlines HC’s interpretation of novelty of plant breeding products. This includes a list of five categories of food products that the regulator has deemed as safe, because their safety is already well characterized (Figure 1).

**FIGURE 1:**
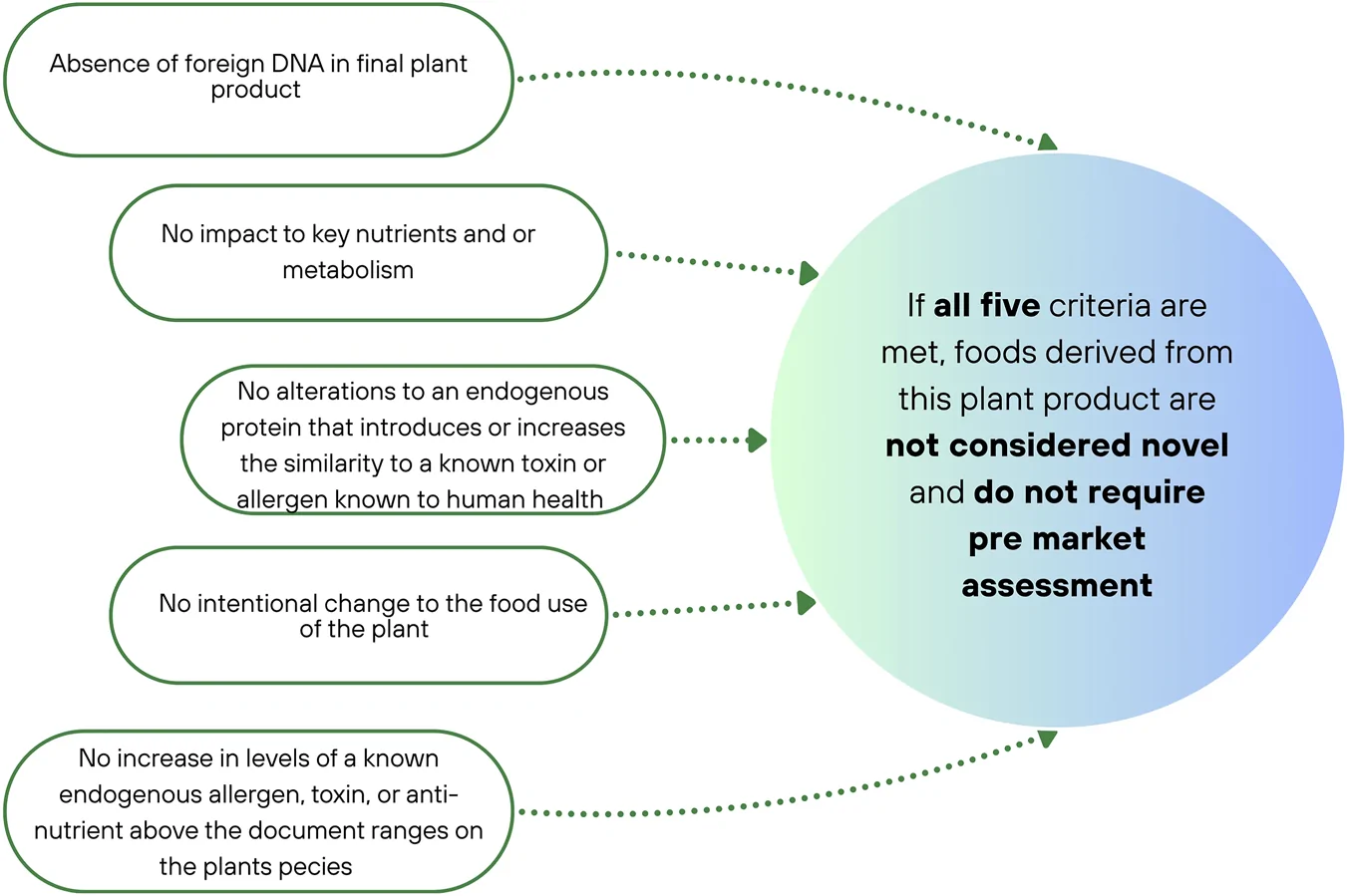
Food product categories where safety is well characterized and considered as safe and not requiring assessment by Health Canada (Health Canada, 2022d).

In parallel, HC also published updated guidelines on the pre-market assessment for food derived from retransformants, added as Appendix II to HC’s Guidelines for the Safety Assessment of Novel Foods. Appendix II outlines the criteria to qualify for the retransformant process and the submission requirements, which has a basis in the scientific literature and regulatory experience (Health Canada, 2022d). This process involves a tiered approach to the information requirements for the assessment of plants developed through the insertion of a DNA sequence identical to another previously assessed novel food (retransformant). Even though these products will be considered novel, they are eligible for a new assessment service standard of 120 calendar days from the day when the complete submission package is received, greatly contrasting with the regular timeline of 410 calendar days for a product that meets the definition of a novel food.

In addition to these measures, Health Canada introduced a voluntary transparency mechanism specifically for genome edited products, whereby product developers notify the department of their novelty self-determination, and upon review the product will be included in a ‘List of non-novel products of plant breeding for food use’ on their website (Health Canada, 2022e). This does not represent a notification for safety assessment or approval. Further details on how this mechanism works are found in the transparency section below, 3.5 Transparency and Communications Processes.

#### 2.3.3 CFIA updates

Oversight of the regulation of novel plant breeding products is performed by the CFIA if these are intended for livestock feed or environmental release (cultivation) in Canada. The unique aspects of these policies are described in the following sections.

##### 2.3.3.1 Environmental release – CFIA Plant Biosafety Office

The CFIA-Plant Biosafety Office published their updated guidelines in May 2023 (CFIA, 2023c). This clarified when a plant would be considered novel and plant breeders and developers would need to seek authorization from the CFIA. The guidance identified two automatic novelty triggers as:

- Expression of commercially viable herbicide tolerance (HT), and/or
- Containing foreign DNA

CFIA clarified that the department considers all plants with new commercially viable HT traits to be plants with novel traits and always require pre-market authorization. For over 30 years, Canada has been the only country in the world to treat herbicide tolerance as an automatic trigger for “novelty” under its product-based regulatory framework, requiring confined field trials, pre-market assessment, submission of herbicide tolerance management plans (HTMPs), and enforceable stewardship conditions – even for traits developed through conventional breeding. With the updates in 2023, the CFIA introduced a significantly streamlined 60-day review process for HT plants that do not contain foreign DNA (e.g., plants developed through conventional breeding, or certain genome edited plants) whereby applicants submit basic information about the trait, and an herbicide tolerance management plan. This contrasted greatly with a standard novelty assessment, which requires extensive data and takes 1–3 years for approval depending on the level of familiarity. This streamlined process improved clarity and reduced regulatory burden which aligned closer with other jurisdictions, but HT continued to be treated as novel by default, meaning Canada still had more regulatory requirements than any other country and represented a unique burden to plant breeders, hindering innovation.

In 2026, the CFIA leveraged their 30 years of experience in regulating HT traits as well as the extensive seed sector stewardship and education activities to make further revisions and remove the automatic trigger for all HT traits (CFIA, 2026c). Under the new direction, when there is no foreign DNA in the plant, developers will determine whether an HT trait is novel based on whether it poses a plausible environmental risk – an outcome expected to be rare. These products will no longer be required to go through the 60-day approval process or be subject to confined field trial requirements. In addition, when approval is required (e.g., when foreign DNA is present) stewardship plans and regulatory conditions will no longer be routinely required and will only be applied where justified by risk. This represents a shift away from prescriptive, trait-based oversight toward outcome-based regulation grounded in scientific evidence and three decades of safe HT crop use while leveling the playing field for Canadian innovation with the rest of the world.

Other than HT plants, the CFIA does not foresee that plants developed using conventional methods would meet the definition of a plant with a novel trait. Nonetheless, it remains the responsibility of plant breeders to consider if the new trait has a plausible pathway to harm the environment and to submit to the CFIA for approval if the product is novel. In determining environmental safety, the breeder/developer should consider the CFIA’s five environmental pillars (weediness potential, impact of gene flow, plant pest potential, impacts on non-target organisms, and impacts on biodiversity) as depicted in Figure 2. An example of how a developer may navigate this system is shown in Figure 3, which outlines a general flow diagram outlining the considerations for environmental risk assessment and the possible outcomes.

**FIGURE 2:**
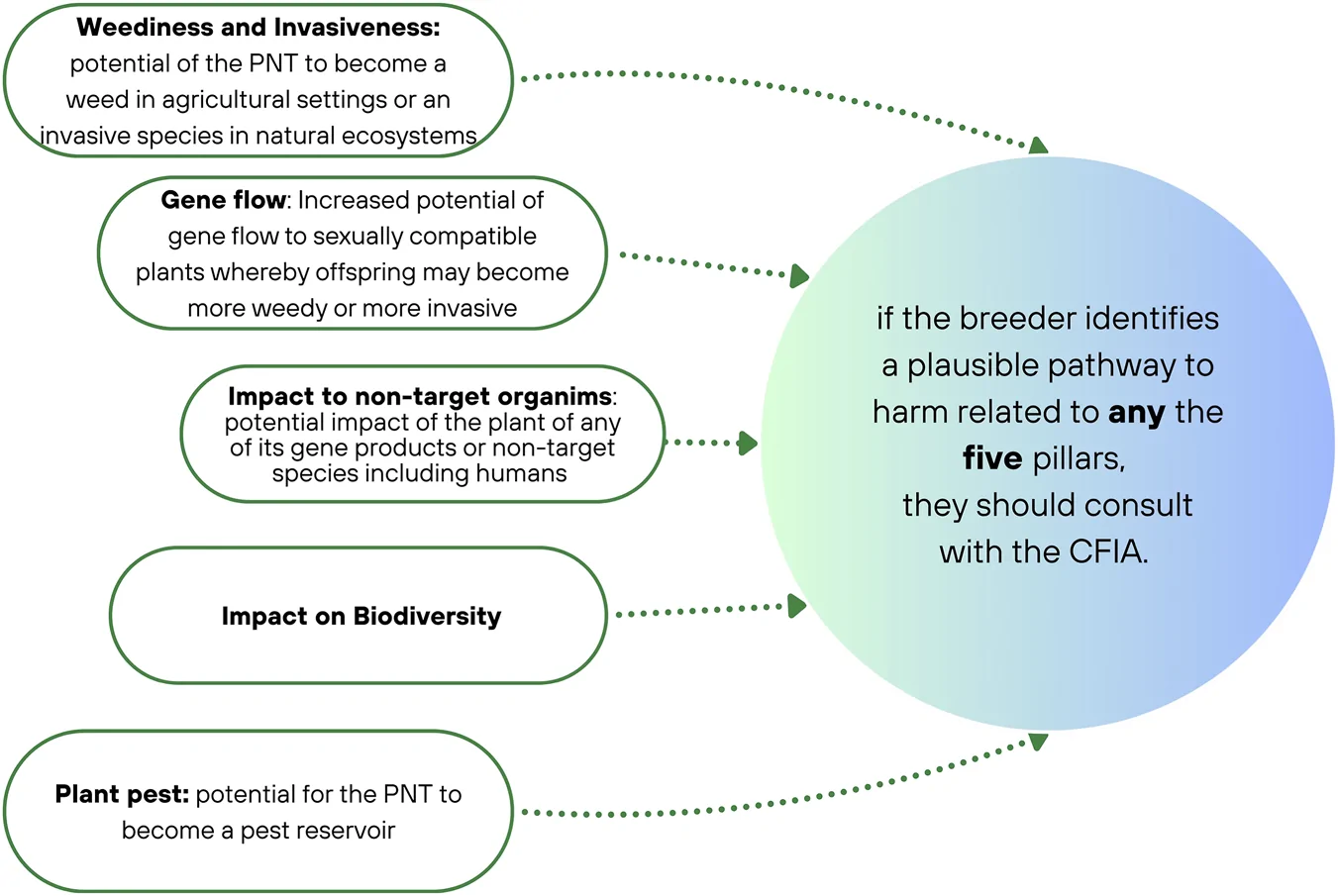
Five pillars of environmental safety taken into consideration by CFIA-Plant Biosafety Office to determine plants with novel traits prior to unconfined release into the environment (CFIA, 2023a).

**FIGURE 3:**
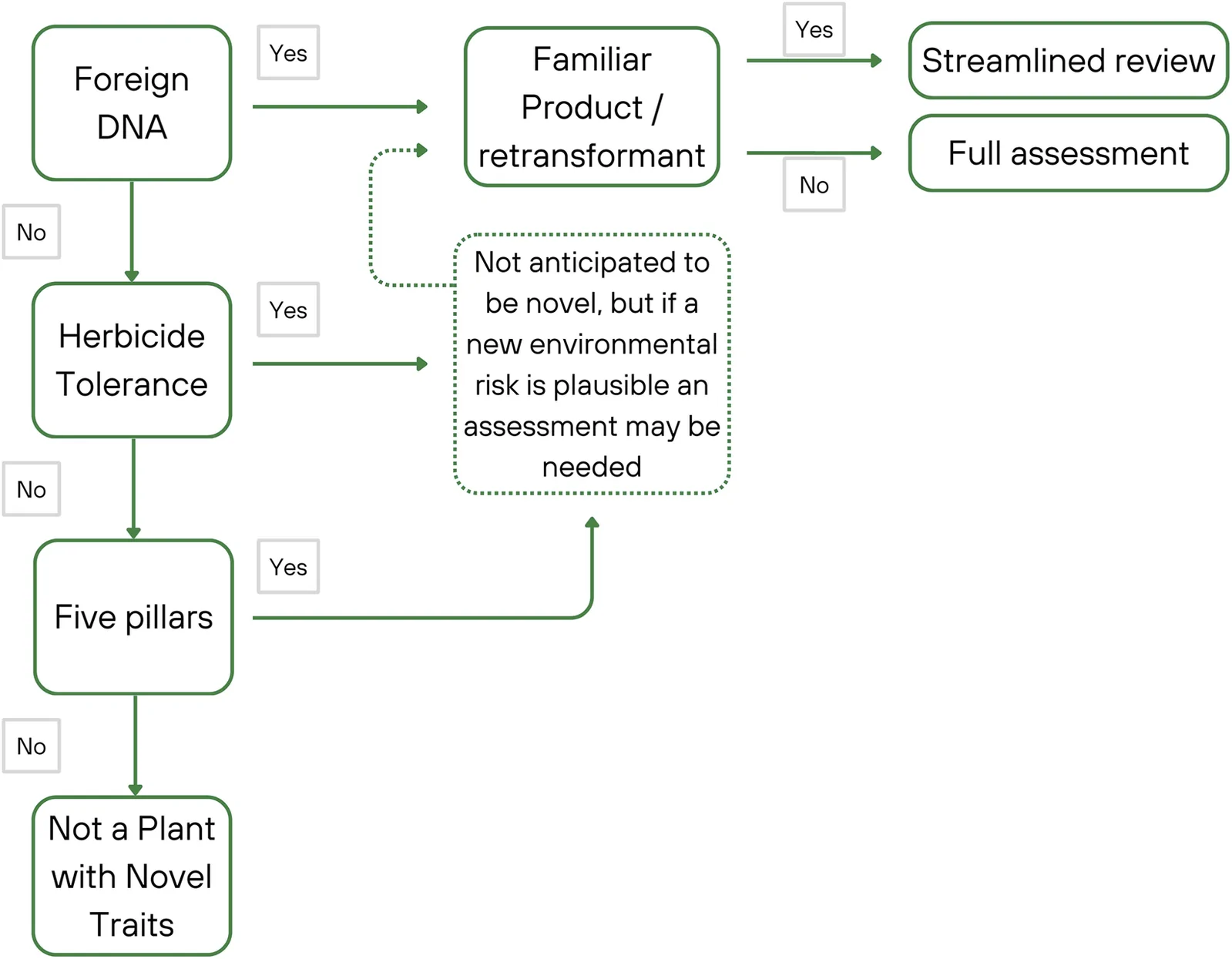
Decision tree to determine if a plant meets the definition of a plant with a novel trait. It is the responsibility of biotechnology developers and plants breeders to assess CFIA’s criteria and notify the authority.

##### 2.3.3.2 Animal livestock feed – CFIA animal feed division

The CFIA-Animal Feeds Division and Veterinary Biologics Division introduced additional guidance in May 2024 to clarify criteria to determine if an ingredient from plant sources constitutes a novel feed. This additional information was added as Appendix III to Chapter 2 – Data requirements for single ingredient approval and feed registration of the existing Regulatory Guidelines (CFIA, 2024b). Whereas the updated CFIA environmental release guidelines and the updated HC guidelines for novel foods are specific to cases of modified plants, the novel feed guidance has a broader scope that captures all feeds from plant sources, and it is not limited to those derived from modified plants. For this reason, the feed guidance is more complex to navigate from the perspective of a developer. The plant biotechnology industry’s interpretation identifies the following three aspects as relevant to the plant breeder:

1. Will the plant or parts thereof be fed to animals?

The first set of considerations are centered around determining if the crop will be used in feed or could end up in feed and if the feed is approved (listed in scheduled IV and V) (CFIA, 2023b). Generally, the industry’s interpretation is that if the crop or any derived ingredients thereof are not listed in schedules IV or V and there is no intention to change the use of the crop to be fed to livestock, novelty is not likely. If there is intent to use as feed, it will require pre-market assessment as a novel feed. If the crop or any derived ingredients are listed in schedules IV or V and the plant has been modified, the presence of a novel trait needs to be assessed.

2. Does the plant/feed contain a novel trait?

Closely aligned with HC’s guidelines on novel foods, according to the CFIA guidance a feed is considered to contain a novel trait when it meets any of seven criteria in Figure 4.

3. Will the feed ingredients have a new claim?

**FIGURE 4:**
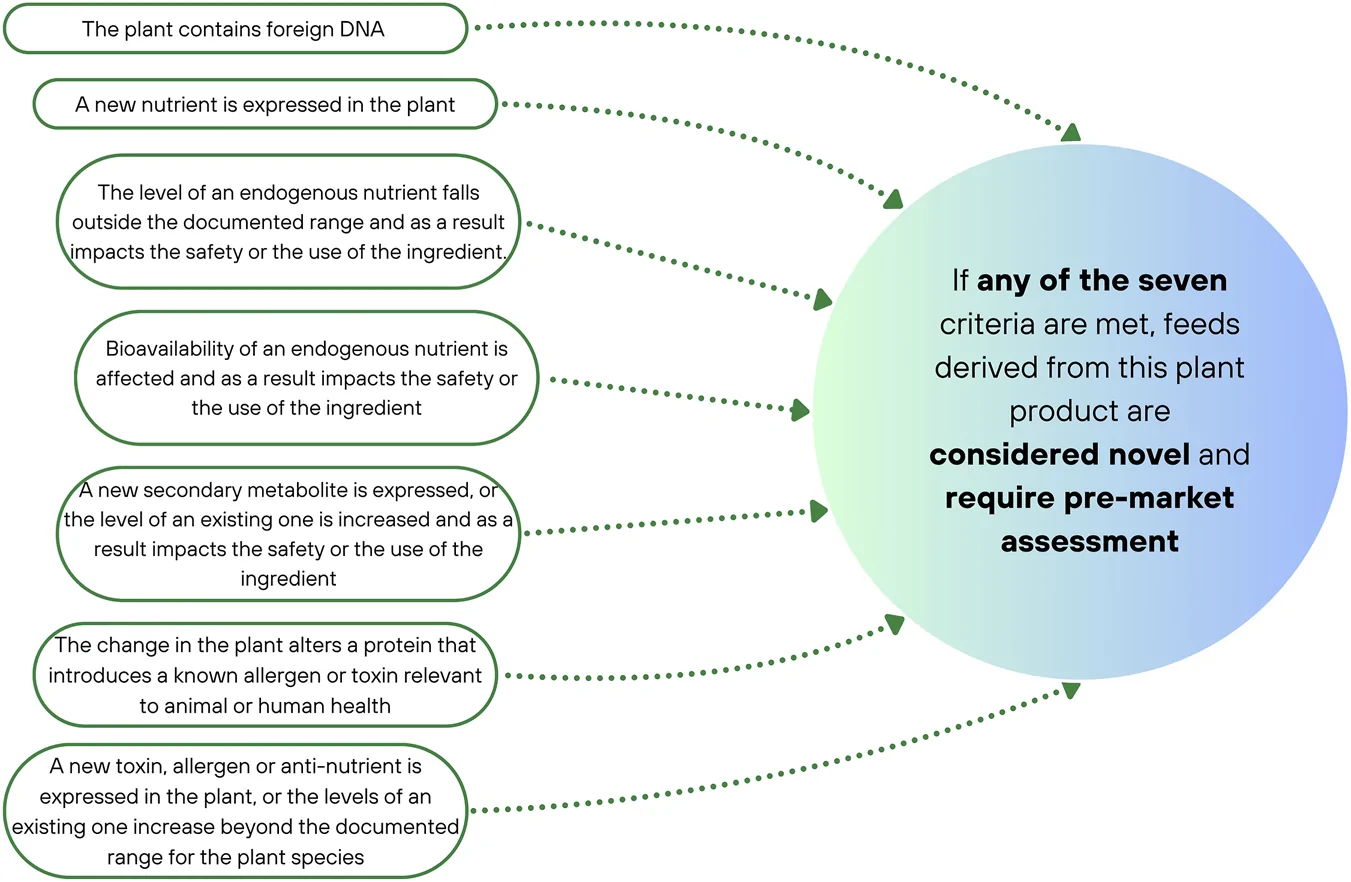
Feed product characteristics that would deem the feed novel hence requiring a pre-market assessment (CFIA, 2024b).

A unique aspect for when a feed needs to undergo a pre-market assessment is when a new claim is made. A claim pertains exclusively to commodities that are sold off-farm for livestock feed purposes and do not extend to seed for planting or commodities to use on-farm. New claims are not permitted to be made unless the feed has already been registered or approved with that associated claim (i.e., Listed under Schedules IV or V).

### 2.4 Historic vs*.* new processes

The updated guidelines provide specific criteria that will facilitate novelty determination by plant breeders and product developers. Paramount to the Canadian regulatory system is the ability of product developers to self-determine if their products are novel or not, something that has been in place since the original regulations were established, and something that continues to be the case after the latest updates. Two additional and important aspects of the new guidelines is the continued implementation of historic and new streamlining policies that add flexibility to the risk assessment process, particularly for retransformants and similar products and the continuation of voluntary mechanisms offered by the regulatory to product developers providing a communication platform to help them through the regulatory process if needed, as shown in the overall comparison of historic and new processes in Figure 5. The additional boxes in step 1 are only intended for genome edited food products that are determined not to be novel. This is a voluntary process not linked to safety but intended to increase transparency for all stakeholders.

**FIGURE 5:**
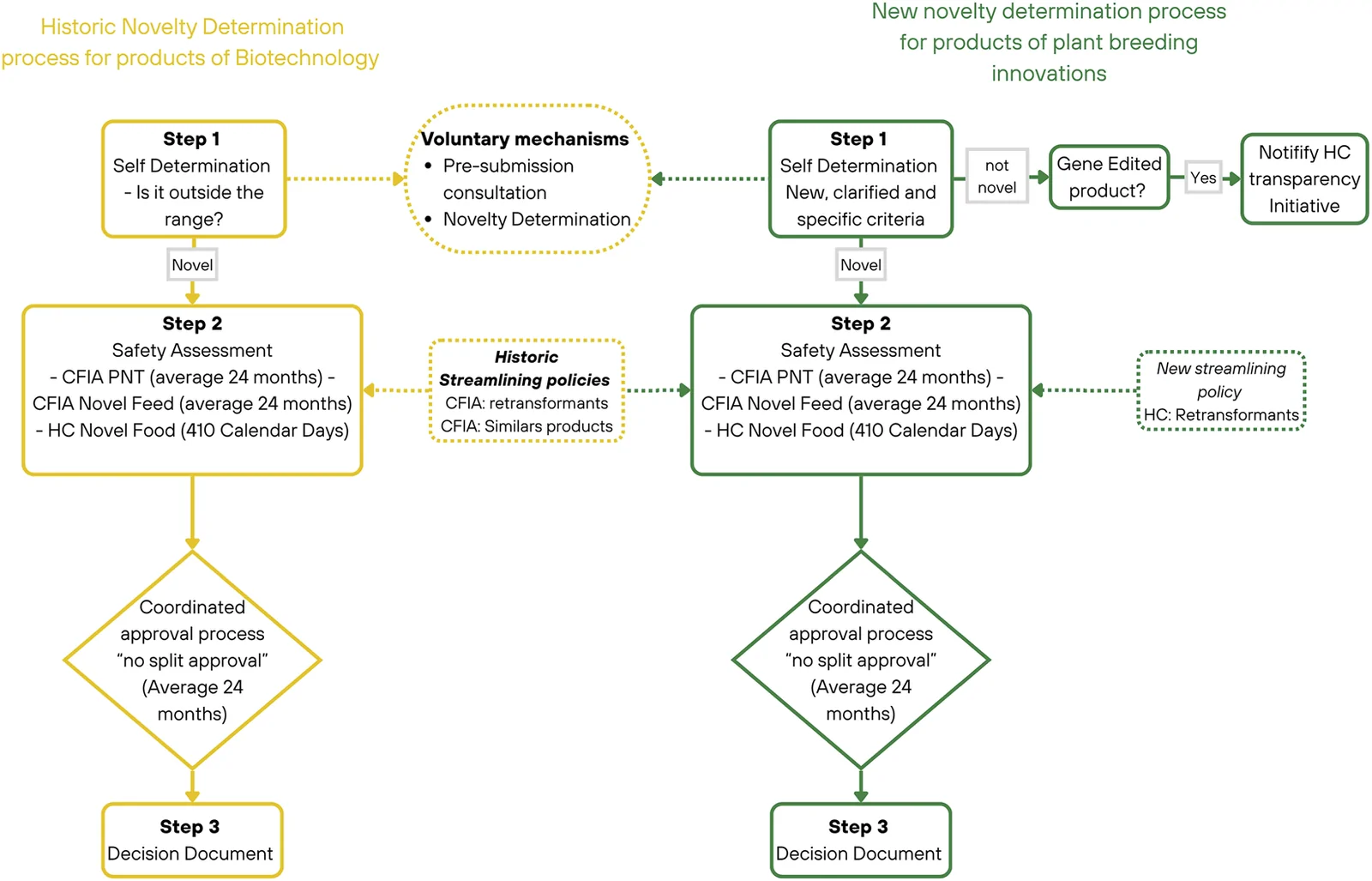
Comparison of a novelty determination process before and after publication of the new guidelines for products of plant breeding. The coordinated approval process ensures that a single, coordinated decision is made for environmental release, human food, and animal feed to prevent unapproved products from entering the market (CFIA, 2019; 2023a; 2024b; Health Canada, 2022d).

A detailed life cycle diagram developed by the Government of Canada outlining the different offices within the departments and their roles can be found on the CFIAs website (CFIA, 2026a).

### 2.5 Transparency and communication processes

In Canada several mechanisms have been established to facilitate transparency and communication for all interested stakeholders related to the regulation and commercialization of biotechnology products. These processes are both government and industry-led efforts that complement each other by addressing different needs identified by various stakeholders (e.g., general public, commodity associations, grain exporters) during the commercialization stages of a product. These are described in the following sections, and a summary is presented in Table 3. In an attempt to centralize information, Agriculture and Agrifood Canada, published a website listing various transparency initiative that enable stakeholders to access information through a single window (AAFC, 2025b).

**TABLE 3:** Summary of mechanisms supporting transparency and communication on regulation and commercialization of biotechnology products in Canada.

Organization	Year started	Name of document/initiative	Information published	Timing	Objective
Health Canada and CFIA (Health Canada, 2026a)	1991	Completed safety assessments of novel foods including genetically modified (GM) foods	Information on approval status and link to summary of information reviewed and opinion	Posted after regulatory decision is made	Provides transparency of what products have been approved and could enter the Canadian market. Aimed at enhancing public trust and regulatory predictability
CFIA (CFIA, 2026b)	Circa 1996	Decision Document	Summary of the information reviewed, safety conclusions, and regulatory decision	Posted after regulatory decision is made	Provide transparency on products that have been reviewed and approved
CFIA (CFIA, 2026d)	Circa 1996	Database of Plants with novel traits (PNT) and novel feeds from plant sources approved in Canada	Information on approval status and links to CFIA decision documents for PNTs and novel feeds	Posted after regulatory decision is made	Provides transparency of what products have been approved and could enter the Canadian market. Aimed at enhancing public trust and regulatory predictability
CFIA (CFIA, 2025a)	2004	Notice of submission	Brief summary of the product and information submitted	Posted shortly after request by developer for a pre-market assessment. Provides public opportunity to provide input or ask questions	Provides transparency on products submitted for review and provides opportunity for public comment
Health Canada (Health Canada, 2022e)	2022	Transparency Initiative	All products developed with gene editing that are not novel are notified to HC and listed in the non-novel list	Submitted at least 90 days prior to commercialization	Enhance public trust and provide Canadians with transparency on the types of gene-edited plant products that may be used in food in Canada
Health Canada (Health Canada, 2026b)	2022	List of non-novel products of plant breeding for food use	Includes list of non-novel foods that were notified to Health Canada through either 1. The transparency initiative or 2. Developer sought a novelty determination	Novelty determinations are typically sought in early product development and posted upon completion; TI notifications are submitted close to commercialization and posted upon submission	Provides transparency of non-novel products, and allows interested parties to verify what products of plant breeding are considered non-novel
Agriculture and Agrifood Canada (AAFC, 2025b)	2025	Transparency – Plant Breeding Innovations	Website consolidating industry and government led transparency initiatives aimed towards genome editing products	Transparency initiatives can be communicated to AAFC for visibility on the website	Provides high-level information on Canada’s regulatory system, and facilitates in a centralized manner easy access for stakeholders to transparency initiatives currently in place
CFIA (CFIA, 2025b)	2025	List of non-novel products of plant breeding for environmental release	List of products where developers requested a novelty determination by CFIA	Novelty determinations are typically requested early in product development and are added to the list upon completion of review	Provides transparency of non-novel products, and allows interested parties to verify what products of plant breeding are considered non-novel
CropLife Canada (CropLife Canada and Seeds Canada, 2021)	2021	Best management practices for launching products of plant breeding innovation in Canada	Highlights the role of developers and the importance of continued and open communication among all value chain stakeholders	Provides guidance for timely and consistent consultations with the value chain, before introducing plant breeding products in Canada	Support timely introduction of plant breeding innovations to the market and minimize trade disruptions
CropLife International & Global Stewardship Group (ETS, 2019)	2019	Excellence Through Stewardship (ETS)	Best practices for commercialization of biotechnology plant products	Educational tools to guide and assist users in developing and implementing organization specific stewardship programs	Provide plant biotechnology industry stakeholders with general stewardship program guidance and specific stewardship considerations
CropLife Canada (CropLife Canada, 2023)	2022	Transparency in plant breeding innovation	CropLife Canada support of various transparency efforts	Ongoing	To promote active engagement in the development and promotion of various transparency initiatives by the plant science industry
Seeds Canada (Seeds Canada, 2025)	2022	Canadian Variety Transparency Database	List of varieties commercialized in Canada with an identification if produced with gene editing and the regulatory status	Posted prior to the variety being commercially sold in Canada	Provide transparency for varieties developed with gene editing that are commercially available in Canada

#### 2.5.1 Government initiatives

##### 2.5.1.1 Database of approved products

The Canadian regulatory system for products of plant breeding is supported by publicly accessible databases maintained by the Canadian Food Inspection Agency and Health Canada that provide transparency regarding regulatory decisions for plants with novel traits (PNTs), novel feeds, and novel foods. The CFIA database of approved PNTs and novel feeds from plant sources compiles regulatory decision documents for products authorized for environmental release and livestock feed use in Canada and also indicates corresponding novel food authorization status by Health Canada. (CFIA, 2026b). This database is searchable by several parameters including crops, traits, company and other parameters. Complementing this resource, Health Canada maintains a publicly available list of approved novel foods and related safety assessments, including foods derived from biotechnology and other novel processes. These databases are updated as products are approved and provide summaries of completed pre-market assessments and “no objection” decisions, supporting transparency, public confidence, and regulatory predictability for developers and stakeholders (Health Canada, 2026a).

##### 2.5.1.2 Decision documents

This was the first transparency process implemented in the mid-90s, as part of the CFIA and HC decision making process. Both departments posted summary documents on their websites explaining in detail the data reviewed and the rationale for reaching their conclusions. Decision documents published by CFIA provide background information describing the product and the results of the environmental risk assessment and the livestock feed use impacts (CFIA, 2023d). Summaries of regulatory decisions by HC concerning novel foods are posted once the product has been determined to be safe for human consumption and are based on a comprehensive review of the information submitted by the applicant (Health Canada, 2026a).

##### 2.5.1.3 Notices of submission

Despite the transparency achieved through the public posting of decision documents, it only allowed for transparency after the assessment (i.e., post regulatory decision). To complement this process, in 2004 the voluntary Notice of Submission project (NOS) was launched to increase transparency and confidence in regulatory processes. Via this process, CFIA posts notices on the NOS website describing a summary of the product and the data submitted by applicants requesting a premarket safety assessment. This is a voluntary process whereby applicants have the option to submit along with their product submissions, dossier summaries for public posting. Through this process, the public is informed about what is being evaluated and has the opportunity to provide input on scientific matters relevant to the submission (CFIA, 2025a).

##### 2.5.1.4 Health Canada list of non-novel products of plant breeding for food use and transparency initiative

With growing application of genome editing in plant breeding, and the likelihood that some of the resulting products will not meet the definition of a novel food, HC introduced a new transparency mechanism to list non-novel products of plant breeding for food use. The non-novel list draws from two sources–the transparency initiative (TI) process and the novelty determination process (Health Canada, 2022e). The TI process was implemented in 2022 alongside the updated guidance for determining novelty and is for food products developed through genome editing that don’t meet the criteria for novelty, Figure 1. (Health Canada, 2022e). The TI invites developers to provide concise information about their product and the basis for their non-novelty determination prior to commercialization of their product. In order to simplify this process, HC created a template which is available to developers upon request. HC reviews the information submitted to ensure completeness and to identify anything that may indicate possible novelty. This process is often referred to as a notification although it does not result in an approval, safety assessment or a novelty determination. Rather, it consists of a review of the product description, and the rationale to support the non-novel status of the product. In a letter to the Minister of Health, CropLife Canada and its members have fully committed to support and participate in transparency mechanisms that enable different farming systems to thrive alongside one another (CropLife Canada, 2023).

Once HC deems the form and information are complete, a summary of the product information is published. This information is free from confidential business information and contains the name of the developer, plant species, product name, intended trait and the mechanism of action, technology use (e.g., TALEN, CRIPSR, Classic mutagenesis) and the intended date of commercialization in Canada as food (Health Canada, 2026b).

Even though it is unlikely that the department’s opinion would differ from the developer’s novelty determination status based on the new guidelines, HC reserves the right to request additional information if there is valid reason to believe that the food may actually meet the “novel food definition”. If novel, the food would have to go through a pre-market assessment.

##### 2.5.1.5 Novelty determination process

This process is intended for any products of plant breeding (including those developed using genome editing or conventional breeding tools) where a proponent is unsure if their product is novel and would like Health Canada to determine if the product meets the novel food definition. Developers submit information on their product and, if this information is complete, Health Canada determines if the product is or is not novel and provides the conclusion in writing to the developer within 60 days. If confirmed non-novel, the product is posted to the list of non-novel products (Health Canada, 2024b). It is important to highlight that if a developer notifies Health Canada through the TI process, they are not expected to submit through the novelty determination process and *vice versa*. If the product is deemed to meet the novel food definition, the food product or ingredient will require a pre-market safety assessment.

##### 2.5.1.6 CFIA list of non-novel products of plant breeding for environmental release

In May 2025 the CFIA launched a list of non-novel products of plant breeding intended for environmental release in Canada. The products in the list are limited to products where the developer has requested a CFIA novelty opinion and has been determined to be non-novel by CFIA regulators, meaning that they are equivalent in terms of their use and safety to those already present in the Canadian environment. The list includes non-confidential summary information on the product such as, name, species, developer, technology used the mechanism of action, and the trait conferred (CFIA, 2025b).

#### 2.5.2 Canadian industry-led efforts for marketplace and value chain transparency

##### 2.5.2.1 Best practices for launching products

One of the main challenges that agricultural biotechnologies face is the lack of globally harmonized regulations and policies, which creates a patchwork of frameworks and asynchronous approvals in different countries. This can delay product commercialization and cause trade disruptions that can have long-term impacts on the competitiveness of cultivation countries such as Canada.

Over the last decade, many countries have developed streamlined genome editing policies that are separate and less onerous than GMO frameworks, however these are not always harmonized and may be cause for confusion. CropLife Canada and several value chain stakeholders created a focused communication framework resulting in CropLife Canada and Seeds Canada publishing the ‘*Best management practices for launching products of plant breeding innovation in Canada’* document in 2021 (CropLife Canada and Seeds Canada, 2021).

This document highlights the important role of developers in continued and open communication among all value chain stakeholders when introducing PBIs into the marketplace. It provides a framework for timely and consistent consultations with the value chain that increase transparency. The scope of the document covers all plant products commercialized in Canada that are subject to a pre-market regulatory process in one or more key trading partners – including streamlined regulatory processes for products developed with genome editing. The guide builds on existing best practices for the commercialization of biotechnology plant products such as product launch policies developed by CropLife International and Excellence Through Stewardship (ETS, 2019).

##### 2.5.2.2 Canadian variety transparency database

While the HC list of non-novel products of plant breeding for food use indicates to the public when a product could enter the market, it does not indicate if or when a genome edited plant variety would be available in Canada. To compliment the HC list and enhance transparency for when seed products are commercialized in Canada, the seed industry launched a list of commercially available seed varieties (Seeds Canada, 2025). The list, hosted by Seeds Canada, obtains data from CFIA’s variety registration records and from voluntary disclosure of its members and partners. The list was designed to include any type of crop, which enables Seeds Canada to encourage developers of crops not subject to variety registration (i.e., horticulture) to provide information for inclusion in the database. This database is meant for products that will be commercially available for purchase by farmers. This database is populated as varieties are posted to the CFIA variety registration database or in advance of commercial sale for varieties not subject to variety registration.

### 2.6 Industry’s views on updated Canadian framework for plant breeding innovations

The updated polices (described above) for PBIs received positive reception from seed industry and agriculture media coverage both in Canada and abroad (Cereals Canada, 2022; 2023; 2024; Western Producer, 2022; 2024; Canola Council, 2023; 2024; CropLife Canada, 2023; Real Agriculture, 2023; 2024). Overall industry comments highlight the improved clarity provided by the updated policies while retaining the product-based regulatory trigger (Lubieniechi et al., 2025). These new policies have also been considered a global model regulatory framework given they are not confined by the number or type of edits like other jurisdictions making them more adaptable as technology evolves such as multiplexing and base editing (Ishii and Ishii, 2022; Ahmad et al., 2023; Cheng et al., 2025; Lubieniechi et al., 2025). Moreover, other countries that have built complex process-based regulatory frameworks have increasingly recognized the need to incorporate a greater product focus to enable adaptivity alongside continuous technological development.

### 2.7 Comparative assessment of regulatory performance

Industry associations representing developers and stakeholders in the plant science sector, such as CropLife Canada, have expressed their commitment to participate in the TI and promote participation beyond its membership. As vested stakeholders in this process, the organization and its members have been closely tracking the implementation of the new guidelines and the transparency initiative to identify if there are any gaps that need to be addressed.

After 3 years of implementation, several genome-edited products have been notified to HC through the TI and listed on HC’s non-novel list (Figure 6). This list also includes products deemed not novel after being submitted to the novelty determination process which predates the TI process and explains the inclusion of products from 2016 to 2021 as well as conventional products.

**FIGURE 6:**
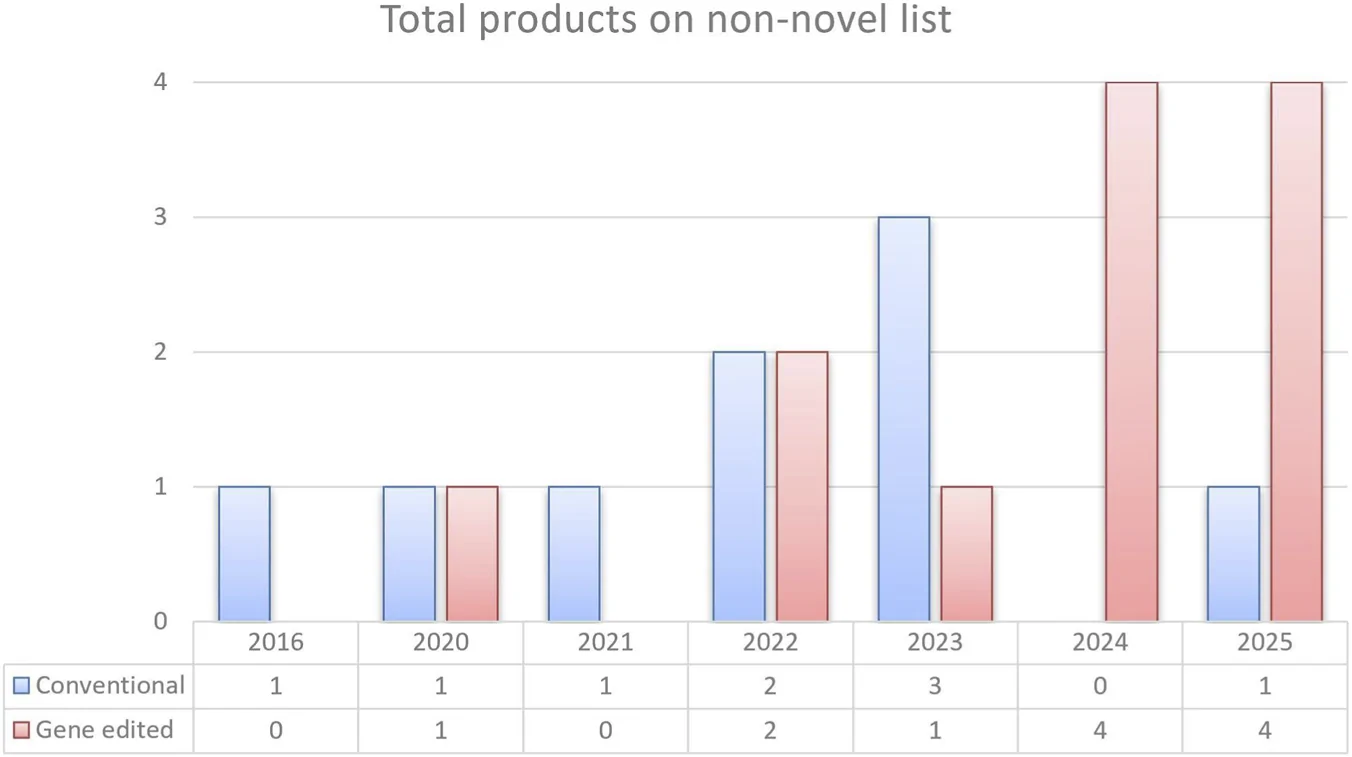
Total notified products in Health Canada’s list of non-novel products of plant breeding for food use by overall breeding method up to May 2026.

From 2016 up to the time of writing (May 2026) the total number of products listed on the non-novel list is 21, out of which 57% (12 total) were developed through genome editing and 43% (9 total) through conventional breeding (Figure 6). Since the introduction of the TI in May 2022 up to May 2026, 17 products have been included in the non-novel list out of which 65% (11 total) were developed through gene editing, and 35% (6 total) through conventional breeding, Figure 6. This is an early indication that product developers support this transparency mechanism.

After nearly 30 years of regulatory approvals and up to May 2026, 165 products have been considered plants with novel traits in Canada by CFIA (CFIA, 2026d). Nearly a quarter of those products (39 out of 165) were developed through conventional breeding, with the remaining 76% corresponding to products considered as LMO (ECCC, 2022).

It is anticipated and has been described elsewhere (Whelan et al., 2020) that greater predictability and streamlined regulatory approaches for PBIs such as genome editing will result in greater diversity in the types of crops and traits developed, and types of developers engaging with the regulatory framework. In the 3 years since the launch of the TI process, nine general categories of traits have been notified (Figure 7) with the majority of the products (38%), corresponding to herbicide tolerant traits, followed by improved harvesting (14%) improved starch content (10%), improved fungal resistance (10%), higher starch content (10%). If contrasted with products on the PNTs Database (Figure 8), we observe that herbicide tolerant PNTs corresponds to 57%, and pest resistance to 26%.

**FIGURE 7:**
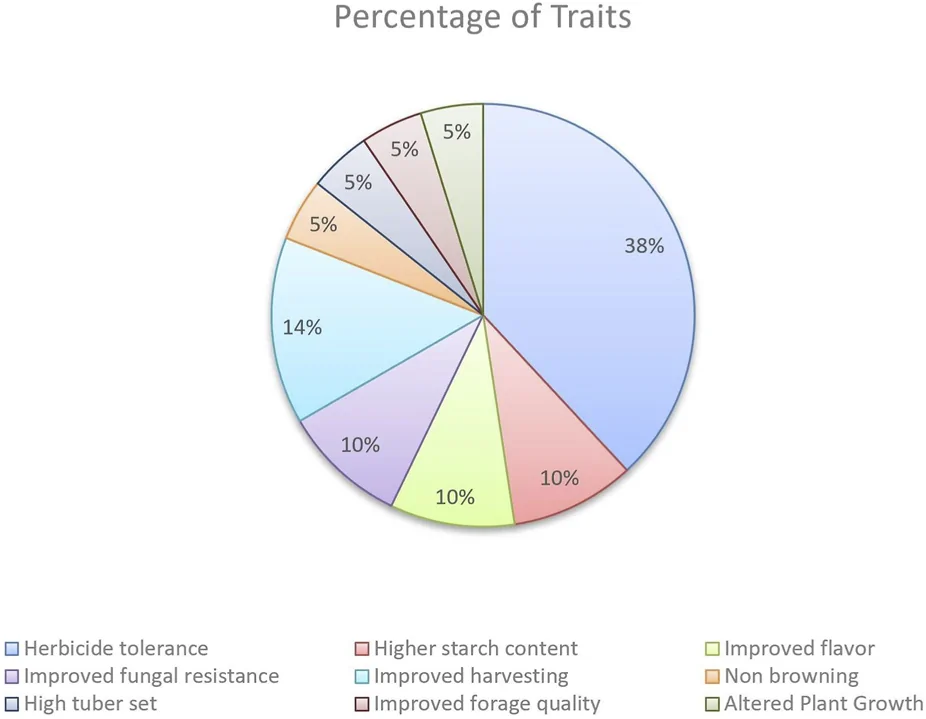
Breakdown of traits in products listed in Health Canada’s non-novel list notified up to May 2026. Improved harvesting includes compact growth, thornless, and longer harvesting time (Health Canada, 2026b).

**FIGURE 8:**
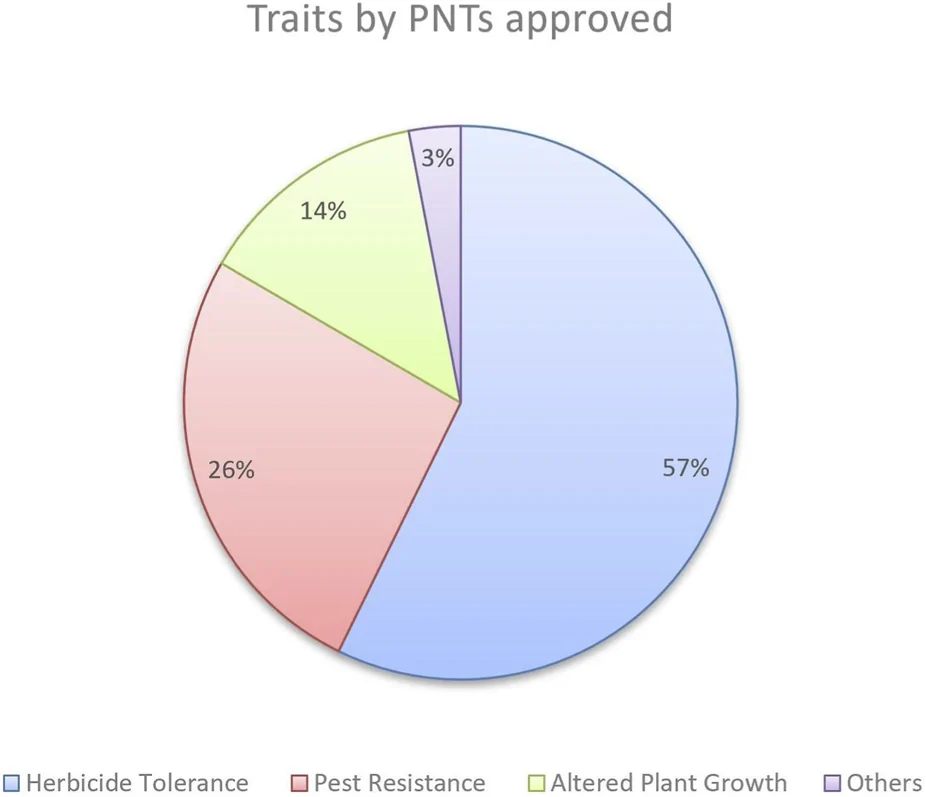
Breakdown of traits in products approved as PNTs in Canada up to May 2026. Other categories includes abiotic stress, optimized fuel production and non-browing traits respectively.

While the TI process was implemented in 2022, the third and final policy needed to clarify the regulatory status of products in Canada was not published until 2024, which was a critical step clarifying the regulatory pathway in Canada and ability for developers to move forward with product commercialization. Furthermore, the global landscape remains uncertain for several countries. We hypothesize this could be the reason why the TI process has not been used more extensively, yet it is still encouraging to see an early snapshot of the greater diversity of traits and crops being notified as non-novel, relative to what was seen for novel traits over a 30-year period.

Another encouraging observation since the publication of the HC non-novel list is that products are notified not only by multinational companies, but also by Small and Medium Enterprises (SMEs) as well as public breeders, with an almost equal amount of non-novel products listed by SMEs and multinationals, Figure 9 (Health Canada, 2026b).

**FIGURE 9:**
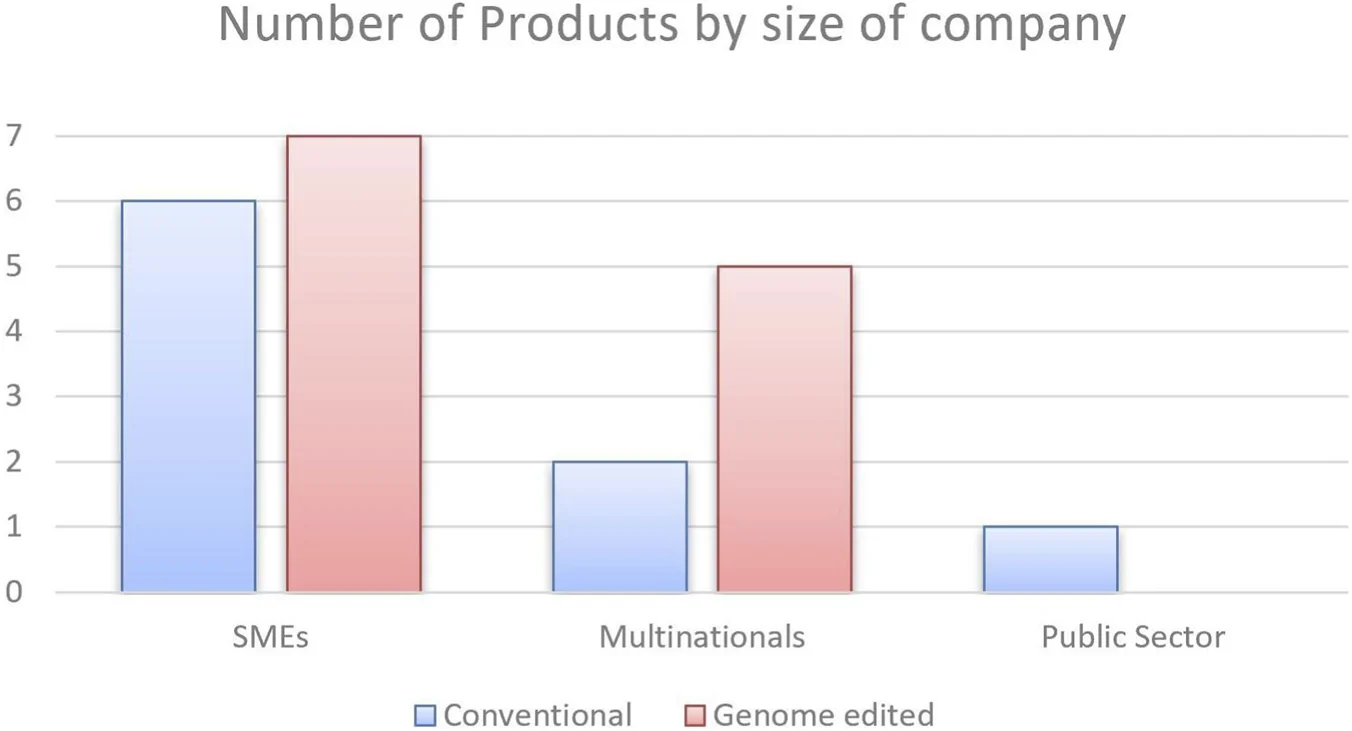
Number of non-novel products by type of entity. Multinationals include new players in the biotechnology space, but with a large agricultural footprint across different jurisdictions.

Comparing only genome edited products, it is encouraging to see a slightly larger number of notifications by SMEs, than large companies. The notified products come from four SMEs and two large companies. The fact that two large companies have almost an equal number of products as four SMEs is not surprising at this early stage as SMEs may have an earlier start in genome editing research and development as reported in studies focused on PBI in the European Union (Jorasch, 2020). This distribution between SMEs and large companies differs significantly with the PNT approval list, which covers 30 years of approvals (1995–2026), as PNT approvals submitted by SMEs correspond to almost 24%, with close to 76% coming from multinationals as seen on Figure 10.

**FIGURE 10:**
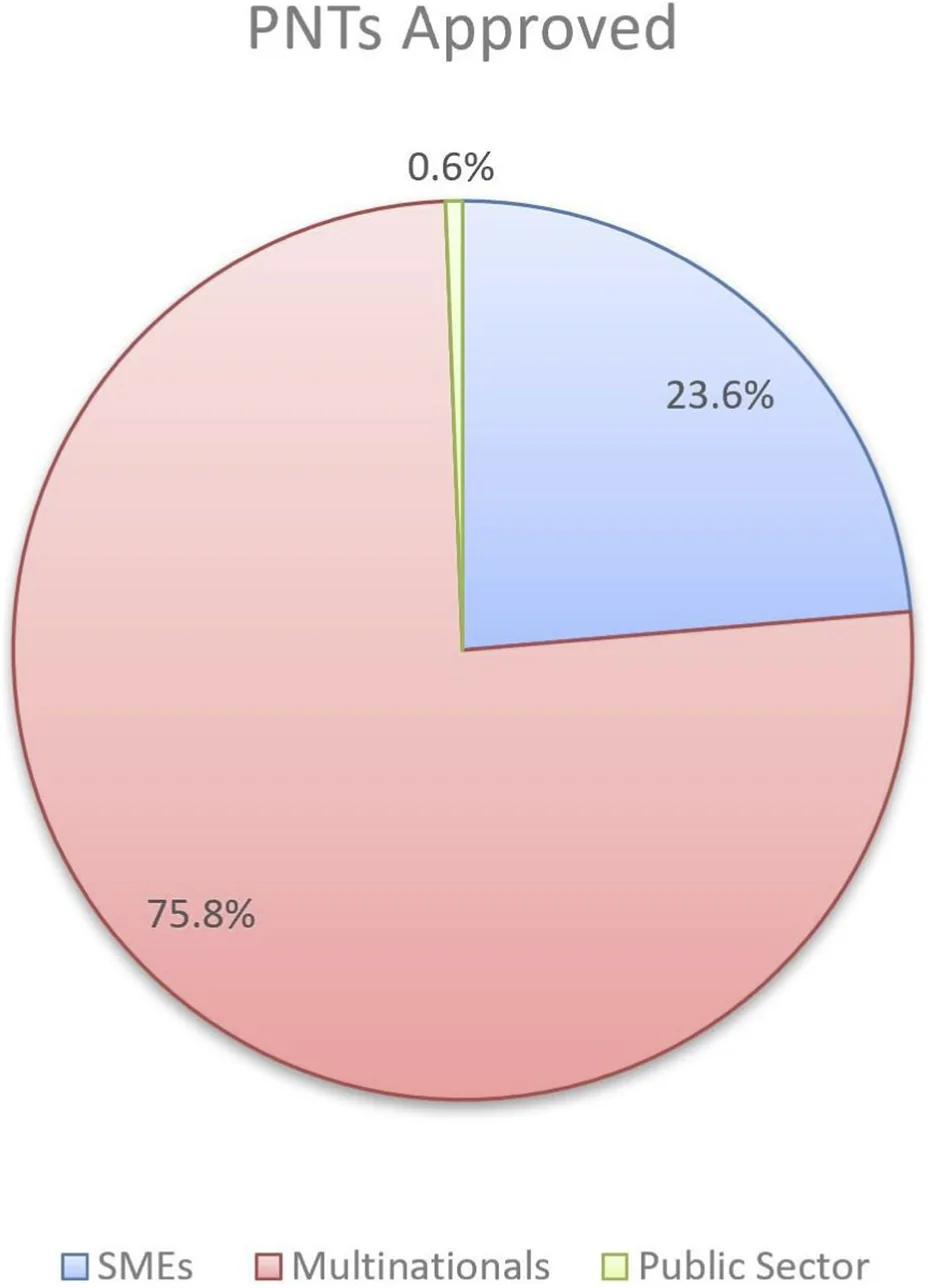
Number of approved PNTs by type of entity. Multinationals include new players in the biotechnology space. The database covers approvals from 1995 to May 2026.

An increased percentage of SMEs notifications has also been observed in countries like Argentina (Whelan et al., 2020). Argentina was the first country to implement a regulatory framework for genome edited products in 2015 (Whelan and Lema, 2015) and has seen the share of SMEs making submissions for regulatory determination increase from 8% to 32%, whereas multinationals’ share decreased from 90% to 9% for the first 5 years since the introduction of the new policy. The biggest contrast when comparing the Argentinian and Canadian cases is the larger increase in participation from local companies and public research institutions in Argentina, from 8% to 59% (Whelan et al., 2020). Several factors may have contributed to this. First, Argentina’s reported figures cover a 5-year period after implementation whereas in Canada only 2 years have passed since all necessary guidance documents were published (the first set in May 2022 and the last in May 2024). Second, although its impact is likely marginal, the Argentinian analysis includes organisms beyond plants, where this paper focuses solely on plants. Finally, Argentina’s regulations require an application for every genome edited line, whereas the Canadian system does not.

Other potential factors contributing to the low number of Canadian public breeders notifying products to date through the TI is their past perception that the regulatory system is an impediment for commercialization of plant breeding products (Smyth et al., 2020; Van Acker et al., 2024) and lack of capital available to agri-food entrepreneurs to scale up technologies domestically (Advisory Council on Economic Growth Potential of Key Sectors, 2017; Van Acker et al., 2024).

## 3 Opportunities for further streamlining of regulatory processes

As the global demand for agricultural products increases, there is opportunity to make Canada a top choice for investment in agricultural innovation, research and development while maintaining Canada’s high standards for health and safety.

The agriculture sector has long been identified as a key sector to increase Canadian exports (Advisory Council on Economic Growth Potential of Key Sectors, 2017). One of the main avenues to support this growth is by modernizing regulations and streamlining approvals that remove barriers to bringing new products to market. Canadian and international stakeholders of the agriculture sector recognize that the government of Canada took an important first step with updating their guidelines for novel foods, novel feeds and plants with novel traits, and in comparison, to other jurisdictions, they are world leading policies that move Canada in the right direction. However, there are still opportunities in other areas where improvements can be achieved.

1. Reduce duplication across government departments.
  - Streamline the approvals for livestock feed. To commercialize novel products of PBIs in Canada requires three separate submissions and reviews covering environmental release, food and feed use. In most cases, these reviews assess substantially similar and often identical scientific information, resulting in unnecessary duplication. Managing three parallel regulatory reviews requires significant time and resources, creating challenges for small and medium-sized developers. In some cases, completion of the feed assessment has delayed product authorization resulting in delays to market. Given Canada is one of the only countries that requires a separate submission and review for livestock feed, there is opportunity to reduce duplication through limiting the need for feed reviews to cases where there is additional scientific value. Specifically, if foreign DNA is the only novelty criteria that is met, assessment by a single agency is sufficient as there are no meaningful feed end point considerations.
2. *Make Canada the most welcoming country for agricultural biotechnology.*
  - Modernize risk assessment process and submission requirements to reflect latest scientific knowledge, regulatory experience, and potential for risk. There are opportunities to leverage the 30 years of Canadian regulatory experience, and flexibility built into the CODEX guidelines to streamline safety assessment processes for novel products. Industry recognizes that there are already existing policies that move in this direction, such as Health Canada’s retransformation policy or CFIA’s “similars” policy (CFIA, 2017; Health Canada, 2022d). Expanding these policies to adopt a more hypothesis-driven risk assessment approach could remove unnecessary default requirements that do not increase safety of products compared to the safety of conventional varieties. For example, non-targeted comprehensive compositional analysis has historically been a default requirement whereas evidence today suggests that requirements for compositional analysis should be hypothesis driven and not be automatically required (Brune et al., 2021)
3. Lead regulatory harmonization efforts.
  - We recognize the efforts that the Government of Canada has undertaken in terms of harmonization and the processes it has implemented to collaborate with the Food Standards Australia New Zealand (FSANZ) in the evaluation of foods derived from genetically modified crops (Health Canada, 2024a; FSANZ, 2025). Opportunities exist to expand these processes to other countries. Continued Canadian leadership in international regulatory fora could further support the development of more aligned, science-based, and predictable oversight approaches for products of modern plant breeding. Actively promoting the scientific principles underpinning Canada’s regulatory framework may help encourage greater international harmonization, reduce unnecessary regulatory divergence, and facilitate innovation, trade, and global access to agricultural technologies.

## 4 Discussion

Canada has implemented a pragmatic science based regulatory framework for the oversight of agricultural biotechnology that has supported the production of safe, high-quality agricultural products. It has some of the most respected regulatory agencies globally, and it has taken bold steps in the past to modernize its systems to keep pace with technological developments. Since its introduction in the 1990s, the framework has demonstrated considerable adaptability as new plant breeding technologies and scientific approaches have emerged. Recent policy updates and transparency initiatives further reflect ongoing efforts by regulators and stakeholders to improve clarity, predictability, and public confidence in the oversight system.

Given the complexity of the framework, involving three regulatory units, with several updated policies in different directives and on different websites, it can be difficult and overwhelming for a developer or a plant breeder or any interested stakeholder to navigate. This policy and practice review brings together the key pieces of legislation and policy elements in a single consolidated reference, while providing historical context and interpretation by experienced industry representatives, to support consistent understanding and interpretation of the policies over time.

Although the new framework is considered to be true to its product-based nature and therefore adaptable to evolving technologies, it will be important for industry and government to monitor emerging technologies such as complex or multiplex genome editing and how they may or should be captured or not captured for pre-market safety assessment. Furthermore, as these technologies are used as part of large plant breeding programs, how a “product” is defined and captured may warrant further consideration e.g., is it a precise edit or the phenotype that defines the product.

Even though the recently updated guidelines clarifying the regulatory approach for PBIs represent significant and important progress in improving regulatory clarity and better support innovation, there are additional opportunities that the Government of Canada can take to further enhance the efficiency and competitiveness of Canada’s regulatory system. As global agricultural challenges and competitive pressures continue to evolve, such as changing climate and changes in trading partners, maintaining a predictable, proportionate, and science-based regulatory environment will remain important to supporting continued investment and innovation within Canada’s agricultural sector. The recommendations in this paper identify practical areas where additional refinements could further strengthen Canada’s position as an agricultural powerhouse.

## Footnotes

1 In the Canadian context *Act* is a written law or statute enacted by Parliament. Regulations are the rules used to carry out the acts. Regulations are more prescriptive than the acts and carry legislative power. Policy refers to how the regulations are being interpreted and implemented providing a baseline for decision-making in a particular issue (Government of Canada, 2017; 2025; 2026).
